# How genomic information is accessed in clinical practice: an electronic survey of UK general practitioners

**DOI:** 10.1007/s12687-020-00457-5

**Published:** 2020-03-03

**Authors:** W. R. H. Evans, J. Tranter, I. Rafi, J. Hayward, N. Qureshi

**Affiliations:** 1 Primary Care Stratified Medicine (PRISM) Group, Division of Primary Care, University of Nottingham, University Park Campus, Nottingham, NG7 2RD UK; 2 I.M.B.E, St George’s, University of London, London, UK; 3 Yorkshire and Humber NHS Genomic Medicine Centre, Chapel Allerton Hospital, Leeds, UK

**Keywords:** Clinical care, Family health, Genomics, Primary health care, Primary care, Family health history, Rare disease

## Abstract

Genomic technologies are having an increasing impact across medicine, including primary care. To enable their wider adoption and realize their potential, education of primary health-care practitioners will be required. To enable the development of such resources, understanding where GPs currently access genomic information is needed. One-hundred fifty-nine UK GPs completed the survey in response to an open invitation, between September 2017 and September 2018. Questions were in response to 4 clinical genomic scenarios, with further questions exploring resources used for rare disease patients, direct-to-consumer genetic testing and collecting a family history. Respondents were most commonly GP principals (independent GPs who own their clinic) (64.8%), aged 35–49 years (54%), worked as a GP for more than 15 years (44%) and practiced within suburban locations (typically wealthier) (50.3%). The most popular ‘just in time’ education source for all clinical genomic scenarios were online primary care focussed resources with general Internet search engines also popular. For genomic continuous medical education, over 70% of respondents preferred online learning. Considering specific scenarios, local guidelines were a popular resource for the familial breast cancer scenario. A large proportion (41%) had not heard of Genomics England’s 100,000 genome project. Few respondents (4%) would access rare disease specific Internet resources (Orphanet, OMIM). Twenty-five percent of respondents were unsure how to respond to a direct-to-consumer commercial genetic test query, with 41% forwarding such queries to local genetic services. GPs require concise, relevant, primary care focussed resources in trusted and familiar online repositories of information. Inadequate genetic education of GPs could increase burden on local genetic services.

**Electronic supplementary material:**

The online version of this article (10.1007/s12687-020-00457-5) contains supplementary material, which is available to authorized users.

## Introduction

Genomics is the study of the whole genome and how it works. Its range of applications is increasing, and currently it plays a role in the diagnosis, prevention and treatment of rare inherited diseases, cancers and infectious diseases. As genomic applications continue to broaden, knowledge of its application will be required in all areas of medicine including primary care (Talwar et al. 2016).

Patients with genetic diseases are common in general practice. Rare diseases, for example, 80% of which have a genetic basis (Institute of Medicine Committee on Accelerating Rare Diseases Research and Orphan Product 2010), may be individually rare but are collectively common with an estimated prevalence of 3.5–5.9% (Nguengang Wakap et al. 2019). General practitioners play a pivotal role in identifying, supporting and managing patients and their families with inherited disorders, and as the largest group of clinicians working in the NHS, with 54,024 licenced GPs in England and Scotland in 2016 (GMC 2018), will be expected to play a greater role in frontline genetic and genomic services.

There are a wide range of genomic educational and information resources available but little evidence of the applicability and educational effectiveness of these resources for primary health-care practitioners (Talwar et al. 2016). Genomic knowledge is limited across medical specialties including primary care, with practitioners often lacking confidence when communicating this information to patients (Talwar et al. 2016; Burke et al. 2006); there is concern that limited knowledge and skills may lead to genomic tests either not being used or misused with consequential harm to patients (Crellin et al. 2019; Burton et al. 2017). Nonetheless, primary care providers perceive genetics as being important (Mikat-Stevens et al. 2014) and have expressed a willingness to enhance their knowledge of genetics (Qureshi et al. 2002).

To ensure resources are appropriate and optimized for GPs, it is important to understand how and where practitioners currently access information around genomics, particularly within the consultation. It is known that GPs access genetic information when they perceive it to be relevant, when they are presented with a clinical problem, rather than proactively ‘just in case’ they come across such a scenario (Mikat-Stevens et al. 2014; Mathers et al. 2010). However, currently little is known about which information is accessed during a consultation.

To identify current and potential educational approaches and sources of genomic information, an electronic survey based on 4 short clinical scenarios was distributed to GPs across the UK. The findings could then be used to provide a platform of information to develop suitable and targeted resources for GPs to manage genomic scenarios in clinical practice.

The objective was firstly to capture which educational approaches are currently used for genomic clinical scenarios:

- Internet resources – including specific web pages
- Intranet resources – local guidelines, resources embedded in the electronic patient record such as referral templates or care pathways
- Local specialist for further information via referral or telephone advice

And secondly to develop a greater understanding of the resources utilized for certain specific genomic scenarios:

- Resources to support clinicians looking after rare disease patient
- Direct-to-consumer genetic testing
- Collecting a family history

Further information was captured on GP’s preferences regarding the format and length of time devoted to genomic education.

## Methods

### Study design and study sample

The electronic survey was designed by a steering group of practising GPs and academics at the University of Nottingham following a structured review of the literature. Previous studies identified the utility of using a scenario-based survey (Hapgood et al. 2002; Qureshi et al. 2002, 2006). Following review by 4 GP colleagues for face validity, the survey was uploaded and shared on the online survey platform JISC (Jisc 2019). A link to the electronic survey was distributed to practising GPs across the UK via a range of methods: an electronic link in the monthly RCGP e-newsletter; local GP information circulars and GP support pages on social media platforms such as Facebook and Twitter. The survey included an introductory page describing the background to the survey and ethics information for participants. The survey was open for 1 year from September 2017 to September 2018.

### Survey questionnaire

GPs were asked what sources they would access for 4 clinical scenarios:

1. A 41-year-old man with possible familial hypercholesterolaemia (FH).
2. A 32-year-old woman concerned about familial breast cancer (FBC).
3. A 28-year-old lady attending for preconception advice, as her sister has a child with Batten disease. Batten disease, also known as CLN3, is a rare fatal inherited degenerative neurological disorder of childhood (Mole and Williams 2001 Oct 10 [Updated 2013 Aug 1]).
4. A patient wishing to discuss a direct-to-consumer genetic test report.

Suggested responses for sources of information included Internet resources such as Clinical Knowledge Summaries (CKS), an online open access evidence base summary maintained by the National Institute for Health and Care Excellence (NICE 2019), and GP notebook, an online reference resource across a wide range of clinical areas targeted for primary care clinicians (General Practice Notebook 2019). Other resources included information held on the intranet, a local closed network with resources often embedded in or linked from the clinical software. This includes local pathways; information held within referral templates; mentor a clinical library linked from the electronic patient record system (EPR) and PRODIGY clinical guidelines and protocols linked from the EPR. In addition, there was the option to liaise with or refer to primary or secondary care colleagues. The survey requested responders to choose all that applied as well giving an option ‘other’ for free text responses.

The survey also asked GPs:

- Their view on genomic educational resources, both the format and length of time they would be willing to spend on keeping up to date
- How they currently approach recording a family history

## Data analysis

Descriptive statistics were used to assess the proportion of responses for questions based on each of the 4 clinical scenarios.

Demographic data gathered included information on participants’ job profile, age, the length of time they had been a GP, practice list size, as a measure of workload, and practice setting (urban/suburban/rural) with suburban practices typically having a wealthier patient population.

## Results

A total of 159 surveys were received. The majority of respondents were GP principals, independent GPs who own their clinics (64.8%), aged 35–49 years (54%), worked as a GP for more than 15 years (44%), practised within a suburban location (50.3%) and worked in a practice with a list size less than 10,000 (49%) (Table 1).

**Table 1:** Characteristics of respondents

	*n* (%)
Personal characteristics	
Age in years	
< 35 years	20 (12.6)
35–49 years	86 (54.1)
> 49 years	53 (33.3)
Job profile	
GP principal	103 (64.8)
Salaried GP	34 (21.4)
Locum	16 (10.1)
GP in training	5 (3.1)
Other	1 (10.6)
Practice location	
Rural	19 (11.9)
Suburban	80 (50.3)
Inner city	60 (37.7)
Practice list size	
< 10,000	75 (49)
10,000–12,000	21 (13.7)
12,000–14,000	23 (15)
14,000–16,000	18 (11.8)
> 16,000	16 (10.5)
Length of time as GP	
< 5 years	27 (16)
5–10 years	37 (23)
10–15 years	25 (15)
> 15 years	70 (44)

Respondents were asked how they had accessed the survey: most respondents had received a link from a colleague (47%); however, some had accessed the survey link via social media platforms (Facebook (24%) and Twitter (2%)). Other methods of accessing the online survey link were stated as the RCGP newsletter (12%), local CCG newsletter (7%) and local research network (8%).

### Care pathways/local guidelines

Care pathways and local guidelines, often available on local intranet systems, were a more frequently preferred resource for the familial breast cancer (FBC) scenario than the familial hypercholesterolaemia (FH) scenario. It was rarely considered a resource for the Batten disease (BD) scenario. Of these intranet resources, local guidelines were the most popular resource for both FBC (69%) and FH (50%) (Table 2).

**Table 2:** Respondents preferred intranet options for genomic information when presented with a patient with a genomic disorder *(Multi-answer: respondents were asked to mark all that applied)*

*Disease*	FH	FBC	BD
*Intranet resource*	*n* (%)	*n* (%)	*n* (%)
Templates	30 (19)	60 (38)	0
Care pathways	43 (27)	78 (49)	0
Local trust guidelines	79 (50)	109 (69)	1 (0.6)
Mentor/prodigy	44 (28)	1 (0.6)	1 (0.6)
Local CCG update	18 (11)	0	0

### Internet searches and resources

NICE Clinical Knowledge Summary (CKS) were a popular resource for both FH (67%) and the FBC (68%) scenarios (Table 3). GP notebook was also frequently chosen as a resource in the FH (64%), FBC (68%) and in response to a follow on question in the FBC scenario, when a specific cancer predisposition gene mutation is found in a close relative (see question 15 Appendix 1) (53%).

**Table 3:** Respondents preferred Internet options for genomic information when presented with patient with a genomic disorder *(Multi-answer: respondents were asked to mark all that applied)*

Internet resource	FH	FBC	Batten disease
	*n* (%)	*n* (%)	*n* (%)
GP notebook	103 (64)	106 (67)	102 (64)
CKS	107 (67)	108 (68)	56 (35)
Online educational modules (RCGP, Doctor.net, HEE, BMJ online)	46 (30)	0	0
Google/Bing	60 (38)	76 (48)	Not asked
NICE guidance	12 (4)	74 (47)	0
SIGN	2 (1)	0	0
Online text book	0	12 (8)	31 (12)
Youtube	0	11 (7)	0
Social media (Facebook, WhatsApp)	2 (1)	29 (18)	0
DOH website	0	0	0
Disease-specific online resource	3 (2) (*SB)	0	80 (50) (BDFA)
General online rare disease resource	0	58 (36)	12 (4)
Red whale	2 (1)	1 (0.6)	0
Wikipedia	0	0	1 (0.6)
Online journal search	0	0	1 (0.6)

*SB (Simon Broome criteria) BDFA (Batten Disease Family Association)

Respondents in all scenarios used Internet search engines (e.g. Google/Bing), with 48% of respondents choosing it for the DTC genetic testing and FBC scenarios. Thirty-eight percent of respondents chose Internet search engines for the FH scenario.

A substantial proportion of GPs had not heard of the 100,000 genome project (65/159 (41%)). The majority of GPs would use search engines (Google/Bing) to access more information on this project (91%), with far fewer accessing information directly from a Department for Health or government web page (19%).

### Local advice support

Local guidelines are an important resource for the two more common genetic disorders and of these highlighted more frequently for the FBC scenario than the FH scenario (Table 2).

Local clinical genetic services or other specialists (e.g. lipid clinics) remain an important source of information in all scenarios. Similarly, the follow on question in the FBC scenario, (question 15 Appendix 1) where a specific cancer predisposition gene mutation is found in a close relative generated a wide range of responses. The most commonly selected responses involved seeking advice from secondary care colleagues (68%).

### Genomic continuing medical education

When asked how GPs would like to keep up to date with genomic medicine, online educational modules were the most popular (70% of GPs expressed an interest in this method); educational materials embedded in the clinical systems (52%) or educational content given in the referral templates (49%) were also popular. When asked how long they would wish to spend on an online learning module 78% of GPs selected either 30 min or 1 h.

Forty-two percent of respondents expressed interest in attending a teaching session by their local CCG, and when asked how long they would wish to attend most (72%) felt that 1–3 h would be sufficient. There was a preference for evening meetings (38%), with a lunchtime update chosen as the second most popular choice (22%).

A proportion of respondents would not attend a continuing medical education (CME) session on genomic medicine (17%). Comments were received on the unrealistic demands on CME across the breadth of medicine. Further comments highlighted the need for ‘just in time’ resources that are well placed, easy to find and from trusted providers. Further comments expressed frustration that there are simply too many resources available, meaning it can be hard to navigate and find the best quality resources, with a further comment requesting ‘access to online, up to date, concise clinical information as and when we need it’.

### Rare disease resources

Intranet resources were not identified as a helpful for the Batten disease scenario (Table 2).

**Chart 1:**
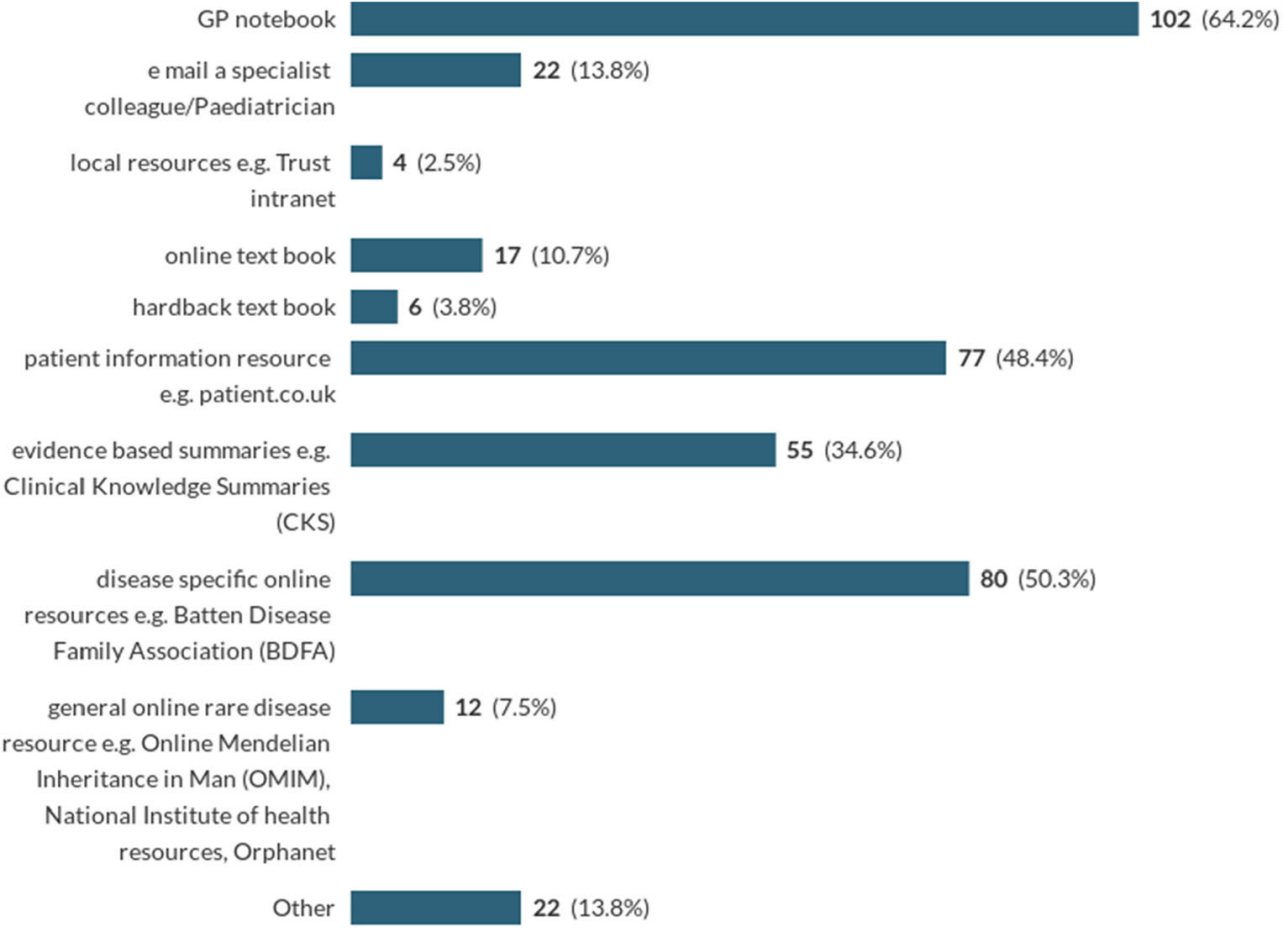
Where GPs would look for information about Batten disease during a consultation *(Multi-answer: respondents were asked to mark all that applied)*

Internet resources were widely used in this scenario with GP notebook identified as the most popular Internet resource (64%) and disease-specific online information such as the Batten disease foundation’s web page (50%) also popular. CKS was less useful than other scenarios (35%) (Table 3). Very few (4%) consider using general rare disease online resources such as Orphanet or OMIM; however, a large number of free text responses simply stated ‘Google’ for information (Chart 1).

**Chart 2:**
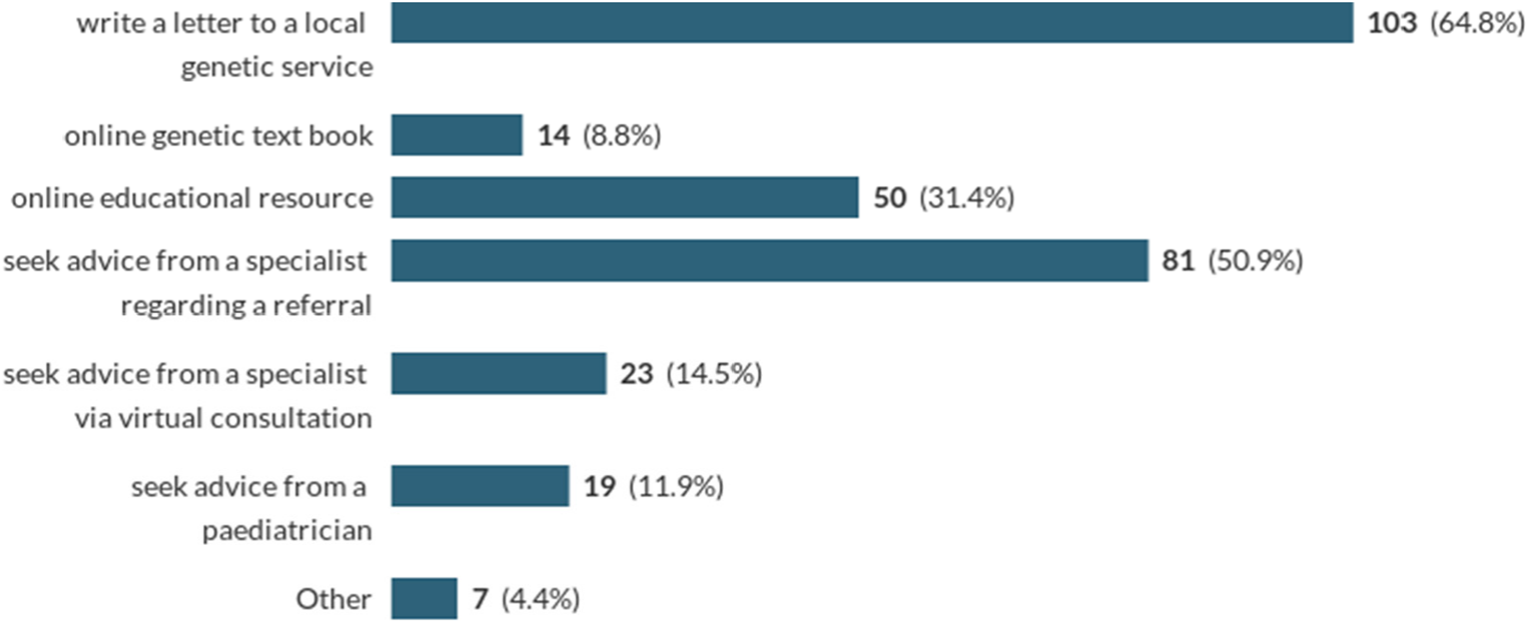
Where GPs access information about a patient’s risk of having a child with Batten disease? *(Multi-answer respondent were asked to mark all that applied)*

Direct communication or referral to local specialist or genetic service was overwhelmingly the most popular response when deciding on the next step to explore preconception testing (Chart 2).

### Commercial direct-to-consumer genetic testing

The survey asked GPs, to pick one resource they would access to gain further information on the value of commercial direct-to-consumer (DTC) genetic testing, most (48%) would use an Internet search engine. Forty-one percent of responses said they would either contact or refer to a local specialist.

A subsequent question asked to pick a single option that would help to support them in reassuring a patient anxious following a DTC test that indicates an increased risk of dementia.

**Chart 3:**
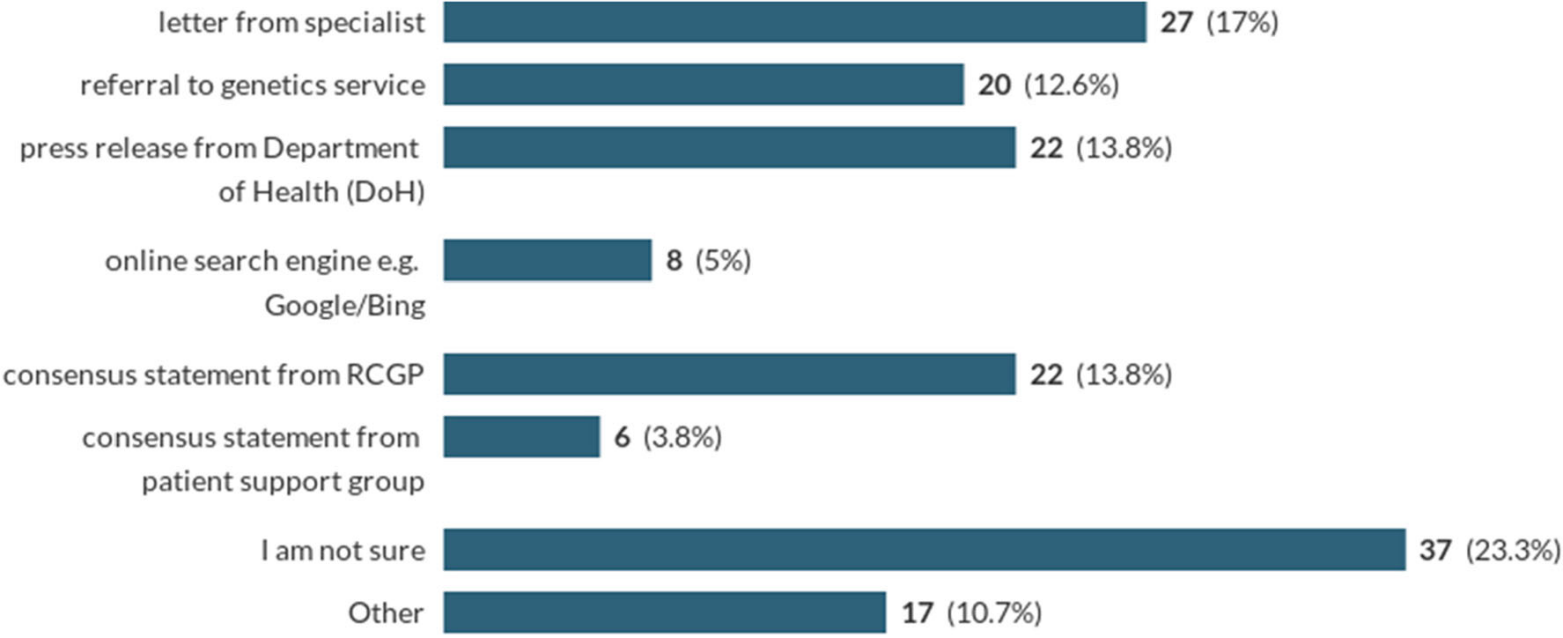
Which further resource would you consider to be the most helpful to support you regarding the value of this commercial genetic testing result and allay patient anxiety? *(Single response question)*

Twenty-three percent of respondents indicated they were unsure, and 30% either indicated a referral to genetics or a letter from a specialist. Statements from organizations such as RCGP (14%) or the Department of Health (14%) were also popular (Chart 3).

### Collecting a family history and managing a patient with a family history of cancer

The vast majority of GPs indicated that they had asked a patient to collect a family history (90%). When asked how they request patients to do this, the most popular options were to ask patients to write a list of relatives (47%), utilize a template from a local genetic clinic (38%) or draw a family tree (31%). (Table 4).

**Table 4:** Respondents preferred options for recording and managing a family history of cancer *(Multi-answer respondent were asked to mark all that applied)*

Resource	*n* (%)
Write a list of relatives	74 (47)
Draw a family tree	49 (31)
Template from local genetic clinic	60 (38)
Complete regional genetics family history form	1 (0.6)
Oxford Cancer genetics referral form	2 (13)
FAHRAS software	1 (0.6)
Internet resources	
Downloadable online tool	9 (6)
Google/Bing	41 (26)
Clinical Knowledge Summaries	108 (68)
GP notebook	91 (57)
Online educational module	
i.e. RCGP, HEE, Doctor.net	58 (36)
Online textbook	12 (8)
Youtube	11 (7)
Intranet resources	
Template	59 (37)
Local guidelines	109 (69)
Care pathways	78 (49)

## Discussion

### Principal findings

GPs surveyed want Internet-based ‘just in time’ resources found in trusted and familiar places, such as GP primary care online educational resources (GP notebook) and national evidence-based resources (NICE Clinical Knowledge Summary (CKS)).

Local specialists and the genetic service remain an important resource for advice and guidance.

Referral pathways and local guidelines, through intranet access, were identified as valuable resources for the FH and FBC scenarios. The popularity of this approach with the FBC scenario may indicate respondents’ knowledge of existing local guidance for FBC and/or familiarity with referral pathways for cancers in general.

The rare disease scenario showed that commonly used Internet resources, GP notebook and CKS are popular places to access information. Additionally disease-specific information such as rare disease charity web pages was also highlighted. Ensuring the accuracy and relevance of information for rare diseases in these widely accessed sites should be ensured. Interestingly, internationally commissioned resources for rare diseases, Orphanet and OMIM, were not used, perhaps a reflection of a general lack of awareness. This echoes the findings in a survey of Belgian family practitioners (Vandeborne et al. 2019). Although the question posed did not have a specific option for search engines (Google/ Bing), free text responses suggest that this is a popular option. Ensuring that appropriate and accurate resources come out at the top of these searches should also be prioritized.

There were mix of responses to the direct-to-consumer genetic testing scenario, with Internet search engines (Google/Bing) as the most popular choice. Such searches were generally more popular when the question posed was less clearly defined or associated with greater uncertainty in how it should be dealt with. Even still, 41% of GPs highlighted that they would either refer or liaise with their local specialist or genetic service. In the context of an increase in the uptake of such testing, this may pose a challenge for genetic services. The most recent data from 23andme suggests that 1.9% of people with reports will attend their GP to discuss the findings, previously estimated at 4% in 2015. If 41% of these consultations lead to a contact with genetic services, this will generate 1500 additional referrals or requests for advice (23andme 2019). Greater guidance on how one should approach such scenarios should be made available to primary care to ensure the appropriate use of clinical genetic services (Horton et al. 2019).

Somewhat surprisingly, given its degree of publicity, a substantial proportion of GPs had not heard of the 100,000 genome project (41%). The majority of GPs would use search engines (Google/Bing) to access more information on this project (91%), with only a relatively small number accessing directly a Department of Health or government web page for more information (19%).

Writing a list of relatives was the most popular method to collect a family history and is a recognized approach to collate this information (Valdez et al. 2010; Qureshi et al. 2009) Also local templates were utilized by some. The mix of approaches amongst respondents may reflect the lack of a single suitable resource or a general lack of awareness of such a resource. Suitable, codable and searchable methods of collecting the details of a family history that is embedded in the electronic patient record system would be of great value.

### Previous research

This study is the first to ask about the current practice of UK GPs regarding genomic scenarios and to explore their attitude and approach to emerging genomic advances.

The preparedness of clinicians to practice genomic medicine has been highlighted to be dependent upon several factors: confidence, perception of the utility of the test, experience, education and the resources available to support them (Paul et al. 2018; Crellin et al. 2019). Previous studies had shown that UK primary care clinicians lack confidence in dealing with clinical genetic scenarios with low levels of genetic training reported in surveys of non-genetic specialists (Burke et al. 2006; Qureshi et al. 2002). Studies have explored the genetic educational needs and preferred approaches in primary care. Calefato et al. (2008) highlighted the genetic educational priorities in primary care physicians in five European countries. A survey of UK GPs has previously demonstrated they valued educational resources that focus on their needs: the recognition and referral of patients and the specific actions they need to take (Burke et al. 2006). Similar priorities were shown in a study of GP’s in the Netherlands with the additional priority: ‘Knowledge of the possibilities and limitations of genetic tests’ (Houwink et al. 2012).

The need to improve genomic education is increasingly acknowledged across the range of specialities with its incorporation into undergraduate and postgraduate curricula and the development of genetic ‘clinical champions’ to develop genomics in their own specialty (Slade and Burton 2016). Mainstream genetic testing is increasingly common in specialties such as oncology, cardiology and paediatrics. How one approaches this is said to be a balance between the push of information from the genetic community, which has frequently been met with resistance by many specialities who could not see the relevance or found it hard to incorporate this into their work streams and the pull for knowledge from physicians when this genomic information has been shown to be relevant and have clinical utility for their patients (Feero and Green 2011).

Our survey demonstrated a preference for online genomic education. Qureshi et al. (2002) highlighted a preference for face-to-face meetings for genetic education, and other educational approaches were offered as options but not specifically online resources. This preference for face-to-face educational meetings has been shown across a range of CME topics; however, in a subset of younger clinicians, online educational materials were more popular with a frequently expressed benefit of ‘anytime and anyplace’ ease of use. Interestingly Braithwaite et al. (2002), in a study aimed at assessing attitudes to a decision support tool for familial cancer, concluded that GPs prefer Internet resources for medical education, as it is both quick and inexpensive. Our finding suggests that for ‘just in time’ genomic information, there is a shift in clinician preference towards online resources available in or between consultations, echoing findings of other studies (Houwink et al. 2012). This is perhaps as one would expect as clinicians’ knowledge and experience of online learning have increased in the years since the earlier studies of GP’s preference.

Vandeborne et al. (2019) surveyed Belgian GP’s rare disease educational needs. They highlighted the following areas: prevention and screening, patient referral, differential diagnoses and rare disease symptoms. Furthermore, their focus group suggested an up-to-date digital platform, freely available in the physicians’ language of choice and validated by numerous rare disease specialists and experts as the most useful resource (Vandeborne et al. 2019). Similarly in a UK survey of the information needs of UK GPs looking after patients with a rare disease, osteogenesis imperfecta, they suggested web-based resources linked to the EPR as the optimum platform (Zack et al. 2006).

### Strengths and limitations

The approach of comparing modalities of information and education across 4 scenarios, each carefully prepared to reflect situations GPs are routinely exposed to, is a strength of this study. It enables the identification of key themes and differences across a range of genomic presentations.

A weakness is the generalizability of the sample. A large proportion of responses were at the request of a colleague; consequently, there was geographical clustering, most notably in West Yorkshire where two authors (WE and JH) are based. This area uniquely has a GPwSI in genetics (JH) which may partly explain a proportion of respondents choosing ‘liaising with a primary care colleague’ for the scenarios: gaining information about the 100,000 genome project and managing patients with breast cancer gene mutations in close relatives. This may not be reflected nationally.

As the study was by open invitation, we could not identify the denominator population; hence, we are unable to calculate response rate. However, the survey was shared nationally through different methods, so a total of 159 responses suggest the response rate was low. This raises further questions about the generalizability of this sample; does the wider population have less interest/knowledge of genomics?

### Key findings for clinical applications

This survey indicates the need for well-placed, concise, online, ‘just in time’ genomic information and educational resources targeted for GPs. These findings are highly relevant as mainstreaming of genomics is implemented. Pathways should ensure opportunities are utilized and maximized for disseminating information, for example, the design of test reports, the linking of laboratory results directly to pathways and resources held in places that GPs are familiar with and accessible options for seeking specialist guidance (Hayward et al. 2017). To ensure this occurs, there must be clear links between the clinical implementation of genomic testing and the provision of educational resources and pathways for GPs (Crellin et al. 2019). Efforts to optimize the design of test reports for non-specialists (Recchia et al. 2019) give a further opportunity for ‘just in time’ education.

It is already acknowledged that the increasing demand for genetic testing exceeds the capacity of clinical genetics departments (Slade and Burton 2016). This study highlights potential further demands with primary care sending additional queries and referrals. In all scenarios, local genetic services were a popular source of information, including inherited cancer predispositions and DTC genetic test queries. Is there capacity within the regional genetic service for this demand? Will new models of working need to be developed to meet this demand and realize the potential of genomic medicine?

Genomics England has a bold ambition to mainstream genomics. The fact that 41% of GPs surveyed had not heard of their flagship 100,000 genome project does makes one question, the success of their approach to ensuring the NHS workforce is on board with this goal.

### Suggested further research

One area of further research, highlighted by the relatively few survey responses and the significant number of GPs unaware of the 100,000 genome project, is an exploration of the relevance of genomics to primary care and the barriers to engagement. The concept of ‘just in time’ resources popular in this survey suggests that the best way to engage the workforce is at the moment of its relevance. Would an appropriate initial step be to share genetic test results, appropriately designed for that audience, with the clinical genetics or other specialty letter to develop a workforce that is both increasingly aware of its relevance and literate in genomics?

Certainly for this survey, further resources could enable its extension to capture a greater number and spread of the primary care workforce. Themes identified in this survey could be explored in greater detail through focus groups or one-to-one interviews, as well as additional genomic scenarios such as dealing with the uncertainty generated by tests results such as variants of uncertain significance or genes with reduced penetrance.

Engagement with secondary care and genomic services for advice remains a popular resource. How this should be done is less clear with a range of responses. Exploring the optimum methods of working and communication would be a further research area of value.

Any educational resources and interventions developed as a result of these study findings should be evaluated for their effectiveness following a robust and structured appraisal process ((EPOC) 2015).

## Electronic supplementary material

ESM 1 (PDF 212 kb)

## Abbreviations

CKS: The National Institute for Health and Care Excellence Clinical Knowledge Summaries
CME: Continuing medical education
DOH: Department of Health
DTC: Direct-to-consumer
EPR: Electronic patient record
FH: Familial hypercholesterolaemia
FBC: Familial breast cancer
GP: General practitioner
HEE: Health Education England
NICE: The National Institute for Health and Care Excellence
RCGP: The Royal College of General Practitioners
