# Supplementary material for: How genomic information is accessed in clinical practice: an electronic survey of UK general practitioners
Source: J Community Genet. 2020 Mar 3;11(3):377–86. doi: 10.1007/s12687-020-00457-5 (PMC7295869; doi:10.1007/s12687-020-00457-5)
Supplement: Supplementary file 1 — (PDF 212 kb) [file 12687_2020_457_MOESM1_ESM.pdf]

How genomic information is accessed in clinical practice: an electronic survey by general practitioners

Page 1: Page 1

BACKGROUND

This online survey has been compiled by a steering group of practising GPs and academics from the University of Nottingham to explore and identify the most effective information needed to appropriately manage patients presenting with genetics and genomic issues.

Genomics is the study of the whole genome and how it works, and has entered all aspects of medicine. There are a range of available educational and information resources that can help GPs to manage patients presenting with genomic issues. So that we can improve understanding of how and where GPs access information about genomics within a consultation, **we would like to invite you to complete this brief survey based on 4 short scenarios of the sort of patients who may consult with you.**

Please read the information sheet on the next page and then tick both boxes to confirm that you:

i) have read the information sheet

ii) are a GP currently working within primary care

Thank you in advance for completing the survey which should take no longer than 15 minutes.

Page 2: Information leaflet

You are invited to take part in a research study and complete an online survey about how you access genomic information. Before taking part, please read the following information carefully and ask us if anything is unclear.

Background

It is estimated that at least 1 in 10 patients seen in primary care has a disorder with a genetic component . General practice plays a pivotal role in identifying patients and families, with General Practitoners (GPs) increasingly expected to play a critical frontline role of genetics services. GPs are reported to use 2 million pieces of information overall to manage patients, although little evidence exists to demonstrate which information is accessed whilst treating patients and which information sources are effective.
It is crucial to understand how and where GPs access information around genomics particularly within a consultation.

What does the study involve?

Completion of a single online survey which takes approximately 15 minutes.

Why have you been chosen?

We are inviting GPs to take part via the Royal College of General Practitioners (RCGP) website.

Do you have to take part?

By completing the survey you are providing consent to take part. You are free to withdraw at any time and without giving a reason.

What are the possible disadvantages and risks of taking part?

There are no anticipated disadvantages or risks.

What if I wish to complain?

You can initially approach the lead investigator (details at the end of this sheet). If this achieves no satisfactory outcome, you should then contact the FMHS Research Ethics Committee Administrator, c/o The University of Nottingham, Faculty PVC Office, B Floor, Medical School, Queen's Medical Centre Campus, Nottingham University Hospitals, NG7 2UH. E-mail: FMHS-ResearchEthics@nottingham.ac.uk

Will my taking part in this study be kept confidential?

All information collected will be kept on a password protected database and is strictly confidential. We will not collect your name and address.

What will happen to the results of the research study?

The results of the study will be published in peer-reviewed publications and presented at conferences. We

will provide anonymised results to the funder who may use the results in future genomic education. You will not be identifiable in any of the reports.

Who is organizing and funding the research?

This study is funded by HEE Genomics Education Programme and approved by the University of Nottingham Medical School Ethics Committee.

Contact for further information: Jennifer Tranter (research assistant) Tel: 0115 846 8041. E mail: Jennifer.Tranter@nottingham.ac.uk

1. I can confirm that I have read the information leaflet ✱ *Required*

- ◯ Yes

1.a. I confirm that I am a GP currently working within primary care ✱ *Required*

- ◯ Yes

Page 3: Scenario 1

Kevin, a 41 year old banker has an appointment following a recent vascular health check.

Although he is following a healthy lifestyle, his fasting cholesterol is excessively raised at 9.4 mmol/L. He is anxious as his mother had a Myocardial Infarction when she was aged 46 years. As there was no secondary cause of raised cholesterol found and he has a strong family history, this is possibly the genetic disorder Familial Hypercholesterolaemia (FH).

(Please now answer the following short questions):

2. If you needed to find further information on this scenario, which **intranet** resource(s) might you use within the practice? (PLEASE TICK ANY BOXES THAT APPLY) ✱ *Required*

- [ ] templates
- [ ] care pathways
- [ ] local guidelines
- [ ] resources within the GP computer system e.g. Mentor, Prodigy
- [ ] local CCG update
- [ ] I wouldn't use intranet resources
- [ ] Other

2.a. If you selected Other, please specify:

3. Which one of the following **internet** resources might you use to find further information in this scenario? (PLEASE TICK ANY BOXES THAT APPLY) ✱ *Required*

- ☐ internet search engine e.g. google/bing
- ☐ short summaries e.g. GP notebook
- ☐ evidence based summaries e.g. Clinical Knowledge Summaries (CKS)
- ☐ general online educational modules e.g. RCGP, Doctor.net, Health Education England, BMJ online learning
- ☐ I wouldn't use internet resources
- ☐ Other

3.a. If you selected Other, please specify:

4. What other method(s) might you consider accessing for information on this scenario? (PLEASE TICK ANY BOXES THAT APPLY) ✱ *Required*

- ☐ social media e.g. Peer support group, WhatsApp, Facebook
- ☐ advice from colleague with additional expertise
- ☐ text books
- ☐ Other

4.a. If you selected Other, please specify:

5. How would you like to access educational materials/keep up to date with genomic medicine? (PLEASE TICK ANY BOXES THAT APPLY) ✱ *Required*

- [ ] local CCG teaching session
- [ ] referral templates provided by GP clinical system
- [ ] educational materials embedded in the GP clinical system
- [ ] online educational module
- [ ] I wouldn't choose educational materials
- [ ] Other

5.a. If you selected Other, please specify:

[ ]

6. How long would you be willing to spend on online educational sessions on genomic medicine? (PLEASE ONLY TICK ONE BOX) ✱ *Required*

- ○ 30 minutes
- ○ 1 hour
- ○ 1-3 hours
- ○ 3-6 hours
- ○ I wouldn't choose online educational sessions
- ○ Other

6.a. If you selected Other, please specify:

[ ]

7. How long would you be willing to spend attending a CME session? (PLEASE ONLY

TICK ONE BOX) ✱ *Required*

- a 30 minute session
- a 1 -3 hour session
- a full day session
- I wouldn't choose to attend a CME session
- Other

7.a. If you selected Other, please specify:

8. When would you wish to attend a CME session on genomic medicine? (PLEASE ONLY TICK ONE BOX) ✱ *Required*

- a local update at lunchtime
- a local update at the weekend
- a local update session in the evening
- I would be willing to travel to attend a CME session
- I would be willing to travel to attend a residential CME session
- Other

8.a. If you selected Other, please specify:

Page 4: Scenario 2

A 32 year old woman attends the practice with concerns about risk of breast cancer for both her daughter and herself, as her sister has recently been diagnosed with breast cancer. Her parents are in good health. She has been advised to return with information about more distant relatives.

(Please now answer the following short questions):

9. Have you ever asked a patient to collect their family history? ✱ *Required*

- ◯ Yes
- ◯ No

10. Please indicate how you would most often advise a patient to collect a family history by ticking ONE box below: ✱ *Required*

- ◯ Not applicable. I haven't asked a patient to collect their family history before
- ◯ write a list of relatives
- ◯ draw a family tree
- ◯ use a downloadable online tool
- ◯ mobile phone App
- ◯ Other

10.a. If you selected Other, please specify:

11. In the future, which of the following internet resources might you use to interpret and manage a patient with a family history of cancer? (PLEASE TICK ALL BOXES THAT APPLY) ✱ *Required*

- [ ] internet search engine e.g. Google/Bing
- [ ] short summaries e.g. GP notebook
- [ ] evidence based summaries e.g. Clinical Knowledge Summaries (CKS)
- [ ] general online educational modules e.g. RCGP, Doctor.net, Health Education England (HEE), BMJ online learning
- [ ] online text books
- [ ] online presentation e.g. YouTube
- [ ] social media e.g. Peer support group, WhatsApp, Facebook
- [ ] I wouldn't use internet resources
- [ ] Other

11.a. If you selected Other, please specify:

12. In the future, which of the following intranet resources might you use to interpret and manage a patient with a family history of cancer? (PLEASE TICK ALL BOXES THAT APPLY) ✱ *Required*

- [ ] templates
- [ ] care pathways

- [ ] local guidelines
- [ ] I wouldn't use intranet resources
- [ ] Other

12.a. If you selected Other, please specify:

13. The patient returns and mentions that her paternal grandmother died from breast cancer when she was 68 years old. Her paternal aunt was invited to take part in the 100,000 genome project which resulted in a test confirming that she carries the mutation in a breast cancer gene called BRCA1. Have you heard of the 100,000 genome project? ✱ *Required*

- ○ Yes
- ○ No

14. Where would you go to find out more information about the 100,000 genome project? (PLEASE TICK ANY BOXES THAT APPLY) ✱ *Required*

- [ ] search engine e.g. Google/Bing
- [ ] online Department of Health (DoH) or government website
- [ ] social media e.g. Peer support group, WhatsApp, Facebook
- [ ] specialist primary care colleague
- [ ] specialist secondary care colleague
- [ ] regional genetics specialist service
- [ ] online medline/journal search
- [ ] Other

14.a. If you selected Other, please specify:

[ ]

15. Where would you access information about managing patients with breast cancer gene mutations in close relatives? (PLEASE TICK ANY BOXES THAT APPLY) *
Required

- [ ] your practice/local intranet resource e.g. desktop App, local health pathway/local information repository
- [ ] Virtual consulting with specialist
- [ ] internet resource e.g. Google/Bing
- [ ] online journal search e.g. Medline
- [ ] online evidence based summaries e.g. Clinicial Knowledge Summaries (CKS)
- [ ] text book
- [ ] GP notebook
- [ ] advice from primary care colleague by phone, e mail or letter
- [ ] advice from secondary care colleague by phone, e mail or letter
- [ ] social media e.g. Peer support group, WhatsApp, Facebook
- [ ] mobile phone App
- [ ] NICE guidelines
- [ ] Other

15.a. If you selected Other, please specify:

[ ]

Page 5: Scenario 3

A 28 year old woman attends as she is planning her 1st pregnancy. She mentions that her sister has recently had a child with Batten disease and would like preconception advice.

(Please now answer the following short questions):

16. How would you access information about Batten disease during the consultation? (PLEASE TICK ANY BOXES THAT APPLY) ✱ *Required*

- ☐ GP notebook
- ☐ e mail a specialist colleague/Paediatrician
- ☐ local resources e.g. Trust intranet
- ☐ online text book
- ☐ hardback text book
- ☐ patient information resource e.g. patient.co.uk
- ☐ evidence based summaries e.g. Clinical Knowledge Summaries (CKS)
- ☐ disease specific online resources e.g. Batten Disease Family Association (BDFA)
- ☐ general online rare disease resource e.g. Online Mendelian Inheritance in Man (OMIM), National Institute of health resources, Orphanet
- ☐ Other

16.a. If you selected Other, please specify:

17. The patient is interested in finding out if she was at risk of having a child affect by Batten disease. You explain that you would look into this and let the patient know the next step. Where would you access this information? (PLEASE TICK ANY BOXES THAT APPLY) ✱ *Required*

- ☐ write a letter to a local genetic service
- ☐ online genetic text book
- ☐ online educational resource
- ☐ seek advice from a specialist regarding a referral
- ☐ seek advice from a specialist via virtual consultation
- ☐ seek advice from a paediatrician
- ☐ Other

17.a. If you selected Other, please specify:

Page 6: Scenario 4

A patient books an appointment to discuss a genetic report. The test was arranged through a mail order service at the local supermarket. She had the test as her mother had breast cancer at 55 years old. The test suggests she doesn't carry the breast cancer gene but has an increased risk of dementia.

(Please now answer the following short questions):

18. Which resource would you be most likely to access to provide information for this patient regarding the value of commercial genetic testing? (PLEASE ONLY TICK ONE BOX) ✱ *Required*

- ◯ information from local specialist
- ◯ CCG intranet resource
- ◯ online search engine e.g. Google/Bing
- ◯ online educational resource
- ◯ Other

18.a. If you selected Other, please specify:

19. Your enquiries confirm that the genetic test has limited clinical value for predicting dementia, however the patient remains anxious. What further resource would you consider to be the most helpful to support your opinion about the value of this commercial genetic testing result and allay the patient's anxieties? (PLEASE ONLY TICK ONE BOX) ✱

Required

- letter from specialist
- referral to genetics service
- press release from Department of Health (DoH)
- online search engine e.g. Google/Bing
- consensus statement from RCGP
- consensus statement from patient support group
- I am not sure
- Other

19.a. If you selected Other, please specify:

THANK YOU FOR COMPLETING THE SURVEY.

WE WOULD BE VERY GRATEFUL IF YOU COULD NOW ANSWER A FEW GENERAL QUESTIONS ABOUT YOURSELF AND PLACE OF WORK ON THE NEXT PAGE.

THIS WILL HELP US TO INFORM THE ANALYSIS OF THE SURVEY.

THANK YOU IN ADVANCE

Page 7: Further information

20. Please tick ONE box that best describes the locality of the practice ✱ *Required*

- rural
- suburban
- inner city/urban

21. Please tick ONE box that indicates the region of the practice where you work ✱ *Required*

- South East England
- London
- North West England
- East of England
- West Midlands
- South West England
- Yorkshire and the Humber
- East Midlands
- North East England
- Central Region Scotland
- Dumfries and Galloway
- Fife
- Grampian
- Lothian
- Orkney
- Outer Hebrides
- Scottish Highlands
- Scottish Lowlands
- Scottish Islands

- Shetland
- Strathclyde
- Tayside
- Trossachs
- Other

21.a. If you selected Other, please specify:

[ ]

22. Please tick ONE box below to indicate the CCG within which your practice is located. (If you are a locum, please tick the box where you work most of the time) ✱ *Required*

- NHS Airedale, Wharfedale And Craven CCG
- NHS Ashford CCG
- NHS Aylesbury Vale CCG
- NHS Barking And Dagenham CCG
- NHS Barnet CCG
- NHS Barnsley CCG
- NHS Basildon And Brentwood CCG
- NHS Bassetlaw CCG
- NHS Bath And North East Somerset CCG
- NHS Bedfordshire CCG
- NHS Bexley CCG
- NHS Birmingham Crosscity CCG
- NHS Birmingham South And Central CCG
- NHS Blackburn With Darwen CCG
- NHS Blackpool CCG
- NHS Bolton CCG
- NHS Bracknell And Ascot CCG

- NHS Bradford City CCG
- NHS Bradford Districts CCG
- NHS Brent CCG
- NHS Brighton And Hove CCG
- NHS Bristol CCG
- NHS Bromley CCG
- NHS Bury CCG
- NHS Calderdale CCG
- NHS Cambridgeshire And Peterborough CCG
- NHS Camden CCG
- NHS Cannock Chase CCG
- NHS Canterbury And Coastal CCG
- NHS Castle Point And Rochford CCG
- NHS Central London (Westminster) CCG
- NHS Central Manchester CCG
- NHS Chiltern CCG
- NHS Chorley And South Ribble CCG
- NHS City And Hackney CCG
- NHS Coastal West Sussex CCG
- NHS Corby CCG
- NHS Coventry And Rugby CCG
- NHS Crawley CCG
- NHS Croydon CCG
- NHS Cumbria CCG
- NHS Darlington CCG
- NHS Dartford, Gravesham And Swanley CCG
- NHS Doncaster CCG
- NHS Dorset CCG
- NHS Dudley CCG
- NHS Durham Dales, Easington And Sedgefield CCG
- NHS Ealing CCG
- NHS East And North Hertfordshire CCG

- NHS East Lancashire CCG
- NHS East Leicestershire And Rutland CCG
- NHS East Riding Of Yorkshire CCG
- NHS East Staffordshire CCG
- NHS East Surrey CCG
- NHS Eastbourne, Hailsham And Seaford CCG
- NHS Eastern Cheshire CCG
- NHS Enfield CCG
- NHS Erewash CCG
- NHS Fylde & Wyre CCG
- NHS Gloucestershire CCG
- NHS Great Yarmouth And Waveney CCG
- NHS Greater Huddersfield CCG
- NHS Greater Preston CCG
- NHS Greenwich CCG
- NHS Guildford And Waverley CCG
- NHS Halton CCG
- NHS Hambleton, Richmondshire And Whitby CCG
- NHS Hammersmith And Fulham CCG
- NHS Hardwick CCG
- NHS Haringey CCG
- NHS Harrogate And Rural District CCG
- NHS Harrow CCG
- NHS Hartlepool And Stockton-On-Tees CCG
- NHS Hastings And Rother CCG
- NHS Havering CCG
- NHS Herefordshire CCG
- NHS Herts Valleys CCG
- NHS Heywood, Middleton And Rochdale CCG
- NHS High Weald Lewes Havens CCG
- NHS Hillingdon CCG

- NHS Horsham And Mid Sussex CCG
- NHS Hounslow CCG
- NHS Hull CCG
- NHS Ipswich And East Suffolk CCG
- NHS Isle Of Wight CCG
- NHS Islington CCG
- return to top
- NHS Kernow CCG
- NHS Kingston CCG
- NHS Knowsley CCG
- NHS Lambeth CCG
- NHS Leeds North CCG
- NHS Leeds South And East CCG
- NHS Leeds West CCG
- NHS Leicester City CCG
- NHS Lewisham CCG
- NHS Lincolnshire East CCG
- NHS Lincolnshire West CCG
- NHS Liverpool CCG
- NHS Luton CCG
- NHS Manchester CCG
- NHS Mansfield And Ashfield CCG
- NHS Medway CCG
- NHS Merton CCG
- NHS Mid Essex CCG
- NHS Milton Keynes CCG
- NHS Morecambe Bay CCG
- NHS Nene CCG
- NHS Newark & Sherwood CCG
- NHS Newbury And District CCG
- NHS Newcastle Gateshead CCG
- NHS Newham CCG

- NHS North & West Reading CCG
- NHS North Derbyshire CCG
- NHS North Durham CCG
- NHS North East Essex CCG
- NHS North East Hampshire And Farnham CCG
- NHS North East Lincolnshire CCG
- NHS North Hampshire CCG
- NHS North Kirklees CCG
- NHS North Lincolnshire CCG
- NHS North Manchester CCG
- NHS North Norfolk CCG
- NHS North Somerset CCG
- NHS North Staffordshire CCG
- NHS North Tyneside CCG
- NHS North West Surrey CCG
- NHS Northern, Eastern And Western Devon CCG
- NHS Northumberland CCG
- NHS Norwich CCG
- NHS Nottingham City CCG
- NHS Nottingham North And East CCG
- NHS Nottingham West CCG
- NHS Oldham CCG
- NHS Oxfordshire CCG
- NHS Portsmouth CCG
- NHS Redbridge CCG
- NHS Redditch And Bromsgrove CCG
- NHS Richmond CCG
- NHS Rotherham CCG
- NHS Rushcliffe CCG
- NHS Salford CCG
- NHS Sandwell And West Birmingham CCG

- NHS Scarborough And Ryedale CCG
- NHS Sheffield CCG
- NHS Shropshire CCG
- NHS Slough CCG
- NHS Solihull CCG
- NHS Somerset CCG
- NHS South Cheshire CCG
- NHS South Devon And Torbay CCG
- NHS South East Staffordshire And Seisdon Peninsula CCG
- NHS South Eastern Hampshire CCG
- NHS South Gloucestershire CCG
- NHS South Kent Coast CCG
- NHS South Lincolnshire CCG
- NHS South Manchester CCG
- NHS South Norfolk CCG
- NHS South Reading CCG
- NHS South Sefton CCG
- NHS South Tees CCG
- NHS South Tyneside CCG
- NHS South Warwickshire CCG
- NHS South West Lincolnshire CCG
- NHS South Worcestershire CCG
- NHS Southampton CCG
- NHS Southend CCG
- NHS Southern Derbyshire CCG
- NHS Southport And Formby CCG
- NHS Southwark CCG
- NHS St Helens CCG
- NHS Stafford And Surrounds CCG
- NHS Stockport CCG
- NHS Stoke On Trent CCG
- NHS Sunderland CCG

- NHS Surrey Downs CCG
- NHS Surrey Heath CCG
- NHS Sutton CCG
- NHS Swale CCG
- NHS Swindon CCG
- NHS Tameside And Glossop CCG
- NHS Telford And Wrekin CCG
- NHS Thanet CCG
- NHS Thurrock CCG
- NHS Tower Hamlets CCG
- NHS Trafford CCG
- NHS Vale Of York CCG
- NHS Vale Royal CCG
- NHS Wakefield CCG
- NHS Walsall CCG
- NHS Waltham Forest CCG
- NHS Wandsworth CCG
- NHS Warrington CCG
- NHS Warwickshire North CCG
- NHS West Cheshire CCG
- NHS West Essex CCG
- NHS West Hampshire CCG
- NHS West Kent CCG
- NHS West Lancashire CCG
- NHS West Leicestershire CCG
- NHS West London (K&C & Qpp) CCG
- NHS West Norfolk CCG
- NHS West Suffolk CCG
- NHS Wigan Borough CCG
- NHS Wiltshire CCG
- NHS Windsor, Ascot And Maidenhead CCG

- NHS Wirral CCG
- NHS Wokingham CCG
- NHS Wolverhampton CCG
- NHS Wyre Forest CCG
- Other

22.a. If you selected Other, please specify:

23. What is the approximate practice list size of the practice where you work? (If you are a locum, please enter details for the practice where you work most of the time)

- <10,000
- 10,000-12,000
- 12,000-14,000
- 14,000-16,000
- >16,000

24. Please indicate you job profile ✱ *Required*

- partner
- salaried GP
- locum
- GP registrar
- Other

24.a. If you selected Other, please specify: *Optional*

25. Please tick the box that indicates your approximate age ✱ *Required*

- <35 years
- 35-49 years
- >49 years

26. Approximately how many years have you worked as a GP? ✱ *Required*

- < 5 years
- 5- 10 years
- 10-15 years
- > 15 years

27. Please could you tick the relevant box below to indicate how you heard about the online survey ✱ *Required*

- RCGP newsletter
- local CCG newsletter
- local research network
- link forwarded by colleague
- Facebook
- Other
- Twitter

27.a. If you selected Other, please specify:

28. Thank you for taking the time to complete the survey. If you have any further comments that you feel will help to inform the survey, please enter your comments below.

Page 8: Thank you for completing the survey
